# A Novel WAC Loss of Function Mutation in an Individual Presenting with Encephalopathy Related to Status Epilepticus during Sleep (ESES)

**DOI:** 10.3390/genes11030344

**Published:** 2020-03-24

**Authors:** Emanuela Leonardi, Mariagrazia Bellini, Maria C. Aspromonte, Roberta Polli, Anna Mercante, Claudia Ciaccio, Elisa Granocchio, Elisa Bettella, Ilaria Donati, Elisa Cainelli, Stefania Boni, Stefano Sartori, Chiara Pantaleoni, Clementina Boniver, Alessandra Murgia

**Affiliations:** 1 Molecular Genetics of Neurodevelopment, Department of Women’s and Children’s Health, University of Padova, 35128 Padova, Italy; mariagraziabellini92@gmail.com (M.B.); mariacristina.aspromonte@studenti.unipd.it (M.C.A.); roberta.polli@unipd.it (R.P.); elisa.bettella@unipd.it (E.B.); alessandra.murgia@unipd.it (A.M.); 2 Fondazione Istituto di Ricerca Pediatrica (IRP), Città della Speranza, 35127 Padova, Italy; 3 Pediatric Neurology and Neurophysiology Unit, Department of Women’s and Children’s Health, University l of Padova, 35128 Padua, Italy; anna.mercante@aopd.veneto.it (A.M.); stefano.sartori@unipd.it (S.S.); clementina.boniver@aopd.veneto.it (C.B.); 4 Developmental Neurology Unit, Fondazione IRCCS Istituto Neurologico Carlo Besta, 20133 Milan, Italy; claudia.ciaccio@unimi.it (C.C.); Elisa.Granocchio@istituto-besta.it (E.G.); chiara.pantaleoni@istituto-besta.it (C.P.); 5 Unit of Medical Genetics, AUSL Romagna, 47522 Cesena, Italy; ilaria.donati@auslromagna.it; 6 Department of Medical Sciences, University of Ferrara, 44100 Ferrara, Italy; cainelli.elisa@gmail.com; 7 Medical Genetics Unit, S. Martino Hospital, 32100 Belluno, Italy; stefania.boni@aulss1.veneto.it

**Keywords:** WAC, ASD, ID, ESES, epilepsy, NGS, gene panel, mosaicism

## Abstract

WAC (WW Domain Containing Adaptor With Coiled-Coil) mutations have been reported in only 20 individuals presenting a neurodevelopmental disorder characterized by intellectual disability, neonatal hypotonia, behavioral problems, and mildly dysmorphic features. Using targeted deep sequencing, we screened a cohort of 630 individuals with variable degrees of intellectual disability and identified five *WAC* rare variants: two variants were inherited from healthy parents; two previously reported de novo mutations, c.1661_1664del (p.Ser554*) and c.374C>A (p.Ser125*); and a novel c.381+2T>C variant causing the skipping of exon 4 of the gene, inherited from a reportedly asymptomatic father with somatic mosaicism. A phenotypic evaluation of this individual evidenced areas of cognitive and behavioral deficits. The patient carrying the novel splicing mutation had a clinical history of encephalopathy related to status epilepticus during slow sleep (ESES), recently reported in another *WAC* individual. This first report of a *WAC* somatic mosaic remarks the contribution of mosaicism in the etiology of neurodevelopmental and neuropsychiatric disorders. We summarized the clinical data of reported individuals with *WAC* pathogenic mutations, which together with our findings, allowed for the expansion of the phenotypic spectrum of WAC-related disorders.

## 1. Introduction

The *WAC* gene (10p12.1; OMIM# 615049) encodes a 647 amino acid protein containing two conserved domains, the WW and the coiled-coil domains. WAC is expressed in fetal stages and has a high expression in specific adult brain regions such as the caudate nucleus, substantia nigra, and thalamus [1]. WAC acts as a versatile adaptor in multiple biological processes under various cellular contexts, both in the nucleus and in the cytoplasm. In the nucleus, WAC regulates the ubiquitination of histone H2B (ubH2B), which plays an important role in chromatin organization during gene transcription [2]. In addition, WAC regulates p53-induced p21 transcription that arrests cell cycle in response to genotoxic stress, and therefore plays a significant role in cell-cycle checkpoint activation after DNA damage [2,3]. In the cytoplasm, WAC localizes to the Golgi apparatus where it activates the VCIP13 deubiquitinase, which is required for Golgi re-assembly [4]. Furthermore, in different conditions, by binding diverse protein partners, WAC may have opposite effects on autophagy regulation. It can positively regulate starvation-induced autophagy, promoting autophagosome formation, or indirectly inhibit autophagy, regulating energy-mediated mTORC1 activity [5,6]. The role of *WAC* in normal cognitive performance is also supported by functional evidence in *Drosophila* [5,7].

The *WAC* gene has been recently associated with a recognizable neurodevelopmental disorder, the WAC-related intellectual disability (ID), also referred to as DeSanto–Shinawi syndrome (DESSH) (OMIM# 616708) [8]. Variable degrees of global developmental delay and/or Intellectual disability ID, behavioral abnormalities, and speech impairment characterize the syndrome. Significant but nonspecific features at birth such as hypotonia and feeding problems are also present. Less frequent features are respiratory and visual problems, and digital anomalies such as brachydactyly and fetal finger pads. Different types of seizures have been reported in about 20% of the affected subjects (febril convulsions, tonic clonic seizures, absence), with a normal inter-critical electroencephalographic EEG pattern. Only one report (published only in Chinese) recently describes a *WAC* patient with an EEG pattern of a status epilepticus during sleep (ESES) [9]. Common dysmorphic features include a squared shaped face with broad/prominent forehead, depressed nasal bridge and bulbous nasal tip, deep set eyes, long palpebral fissures, and wide mouth with a broad chin. To date, a detailed description of the clinical phenotype has been reported only for 20 individuals with *WAC*-related ID [7,8,9,10,11]. All of them, except for one family with presumed parental gonadal mosaicism, have de novo pathogenic loss of function (LoF) variants. The variable degree of ID associated with behavioral problems as well as the non-specific/coarse facial appearance found in patients with *WAC* mutations have significant similarities with other neurodevelopmental disorders. Some *WAC* patients had a suspected clinical diagnosis of Prader Willi (PWS), Smith–Magenis (SMS), Angelman (AS), and Pitt Hopkins (PHS) syndromes [7,8]. This makes it difficult to establish or even suggest a specific clinical diagnosis of WAC-related ID. Thus, identifying new variants and better characterizing individuals with previously described variants in the *WAC* gene will further our understanding of the genotype–phenotype correlation and help to highlight the most peculiar features and the amplitude of the spectrum of WAC-related ID.

Using a targeted gene panel, we sequenced 630 individuals with variable degrees of ID with or without behavioral problems; among them, 70 were negative in the (Small Nuclear Ribonucleoprotein Polypeptide N) SNRPN methylation test as well as in the sequence analysis of genes associated with conditions sharing clinical features with DESSH, such as *RAI1*, *UBE3A*, or *TCF4*.

We identified three *WAC* LoF mutations in three unrelated individuals. Two of them were carrying previously reported WAC mutations, had a moderate ID, and presented different features associated with the WAC-related disorder. The third individual presented focal epilepsy with a typical EEG pattern of status epilepticus during slow sleep (ESES), which has been reported once in this condition [9]. He inherited the novel *WAC* mutation from his father, found to have a mosaicism, that was evaluated for his cognitive neuropsychological profile.

We demonstrated that the novel *WAC* mutation we identified causes a splicing alteration and re-evaluated the cognitive neuropsychological profile of the transmitting mosaic father to investigate the possible pathogenic role of mosaic variants on neurodevelopmental disorder NDDs genes in adult’s neuropsychiatric features.

We compare the clinical findings of these two individuals with those reported in the literature and discuss the co-occurring ESES in our patient and its possible significance in the context of a WAC-related phenotype.

## 2. Materials and Methods

### 2.1. Cohort Description

Patients were referred from clinicians of 17 Italian public hospitals with a diagnosis of non-specific neurodevelopmental disorder, after excluding (Comparative genomic hybridization) CGH array alterations and *FMR1* mutations. Clinical data were collected with a standardized clinical record describing family history and clinical phenotype (auxological parameters, physical features, neurological development, cognitive and behavioral profile) or presence of associated disorders. Data from electroencephalograms (EEG) and brain magnetic resonance imaging (MRI) were also collected when available. The Wechsler Intelligence Scale for Children-IV WISC-IV psychometric test was used to evaluate the intellectual ability of Patient 1. The adaptive functioning of Case 3 was assessed by the Italian adaptation of the Vineland Adaptive Behavior Scales (VABS) interview, administrated to the parents (Supplementary Table S1). VABS interview assesses adaptive behavior at different developmental levels from birth through adulthood. Several domains were evaluated, yielding index scores for socialization, communication, daily living, and motor skills. The father of Case 3 was examined using a neuropsychological battery of tests, validated for the Italian population, which evaluates abstract reasoning, language, memory, attention, executive functions, and visual-motor abilities (Esame Neuropsicologico Breve 2; ENB-2) [12].

Written informed consent was obtained from the patient’s parents or legal representative. This study was approved by the Local Ethics Committee, University-Hospital of Padova, Italy (protocol number 3566/AO/15).

### 2.2. Gene Panel Sequencing

DNA was extracted from peripheral blood using the Wizard genomic DNA Promega Kit (Promega Corporation). DNA from oral mucosa cells was extracted using the QIAamp DNA Mini Kit (Qiagen, Hilden, Germany). Targeted sequencing was performed using a custom gene panel of 74 ID-ASD genes as previously described in [13]. The same approach was used to perform target sequencing of a 31 epilepsy gene panel in Patient 3; the list of genes is available upon request. Briefly, multiplex PCR-based primer panel was designed with an Ion AmpliSeq Designer (Thermo Fisher Scientific, Waltham, MA, USA) to amplify all the exons and flanking regions (10 bp) of WAC. DNA libraries were prepared and enriched using the Ion AmpliSeq Technology (Thermo Fisher Scientific) and sequenced with the semiconductor Ion PGM platform (Thermo Fisher Scientific). Raw data were processed with the Ion Torrent Software Suite, and related plugins. Variants were annotated using ANNOVAR and an in-house pipeline to calculate the frequency of the variant in the internal database. Rare intronic or coding variants flanking splice sites were evaluated for possible effects on splicing by Human Splicing Finder (HSF) [14]. Candidate variants were validated and segregated in the family using Sanger sequencing. Confirmed variants have been submitted to the Leiden Open Variation Database LOVD v.3.0 [15].

### 2.3. Transcript Analysis

RNA was extracted from peripheral blood leukocytes using the PAX gene Blood RNA Kit (PreAnalytiX GmbH, Hombrechtikon, Switzerland), according to the manufacturer’s instructions. Total RNA was reverse transcribed with the SuperScript ^TM^II Reverse Transcriptase (Invitrogen, Life Technologies, Van Allen Way, Carlsbad, California, CA, USA ) using random primers, according to the manufacturer’s instructions. Complementary DNA was then amplified using a primer set designed to amplify exon 2–5; forward primer: CAGTGACAGCACAGGTCACA, reverse primer: CCACTGGCAAACCCACTAGT. The size of the *WAC* PCR product was 293 base pairs. Both wild type and aberrant transcripts were extracted from agarose gel bands and Sanger sequenced. Variant nomenclature follows Human Genome Variation Society HGVS standards using reference sequences NM_016628.4 and NP_057712.2.

## 3. Results

### 3.1. Clinical Assessment

For the three individuals carrying pathogenic *WAC* mutations, the clinical features are summarized in Table 1 and compared to the phenotypic features from other WAC patients reported in the literature. A detailed description is presented for each proband in the following paragraphs from the less to more severe phenotype.

#### 3.1.1. Family 1

**Figure 1:**
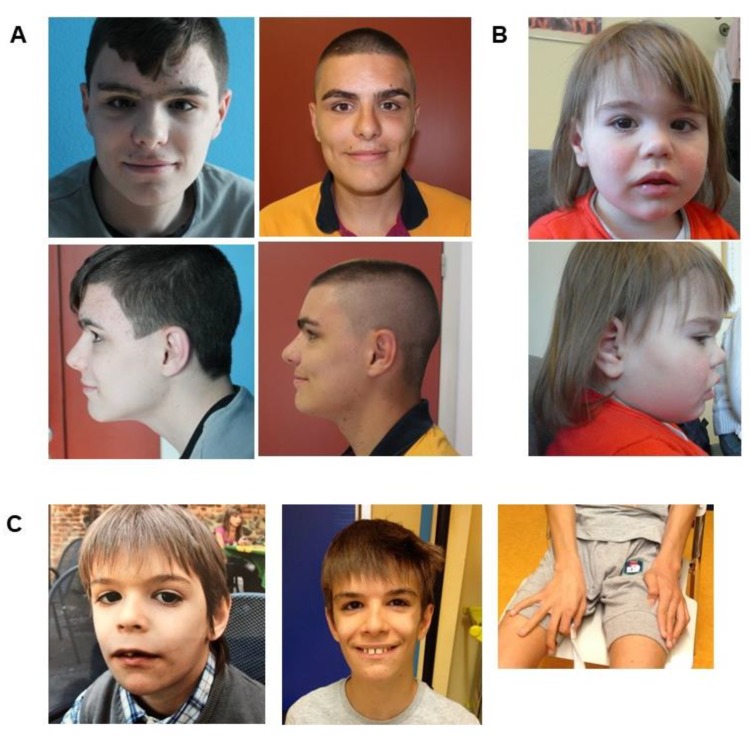
Dysmorphic features of patients with WAC mutations. (**A**) Patient 1 at the age of 14 (first column) and 16 years (second column). Note the protruding supraorbital ridges with bushy eyebrow and long eyelashes, deep-set eyes, bulbous nasal tip, short philtrum, and wide mouth. (**B**) Patient 2 at the age of three. Note: strabismus, short nose with markedly anteverted nares, upper lip with marked cupid bow. (**C**) Patient 3 at the age of eight (first column) and 17 years (second column). Note the square-shaped face with broad forehead, a broad nasal bridge, deep-set eyes with long down slanting palpebral fissures, long eyelashes, bushy eyebrows with mild synophrys, flat philtrum, and thin upper lip. The photo of the hands (bottom) shows the long fingers and his stereotypies consisting of rolling objects and toys.

Patient 1 is a 16-year-old male born at 40 weeks of gestation from non-consanguineous Italian parents. The patient’s mother was 25 and the father was 30 years of age. He was born at term via eutocic delivery after an uncomplicated pregnancy. His birth weight was 3.750 kg (50–75th centile and his length was 51 cm (75–90th centile). His physical growth was regular, but he manifested with psychomotor delay and major language impairment. The child sat independently at seven months and walked at 16 months of age; he started babbling after 12 months of age and said his first words after 24 months. He reached sphincter control at about three years. At the last examination, at age 14, his cognitive and behavioral profile showed moderate ID with a persisting severe expressive language impairment. His intellectual quotient (IQ) as assessed using the WISC–IV scale was 40. He presented a severe speech disorder characterized by stuttering, speech dysfluency, articulation difficulties, and use of a poor and simple vocabulary. His neuropsychological profile highlighted communication and executive function difficulties, short attention span, and behavioral problems including verbal inhibitory control problems. Nonetheless, he was relatively collaborative. No sleeping problems were reported, and an autism diagnosis has not been made. No feeding and gastrointestinal problems were ever observed. He showed a mild *pectus carinatum* and moderate craniofacial dimorphisms; protruding supraorbital ridges, bushy eyebrows, deep-set eyes, bulbous nasal tip, short philtrum, and wide mouth (Figure 1A). Neurological examination was normal, except for a mild global dyspraxia and fine motor skills impairment. He never manifested seizures; EEG and brain magnetic resonance imaging (MRI)MRI were normal. His initial diagnostic examinations included a complete metabolic workup, conventional karyotyping, and Comparative genomic hybridization (CGH) CGH array (40 kb resolution) as well as Fragile X testing, which were all negative.

#### 3.1.2. Family 2

Patient 2 is a 7-year-old female born from non-consanguineous parents with no familial history of developmental delay. The mother, who recently died, was treated during pregnancy with neuroleptics and mood stabilizers due to an unspecified psychiatric disorder. The child was born at 32 weeks of gestation by caesarian section due to the maternal condition; birth-weight 1400 g (10th centile), length 44 cm (25–50th centile), CC 30 cm (25–50th centile), Apgar score 6/9; she had a patent arterial duct and manifested respiratory distress, and sucking difficulties during the first weeks of life. Her psychomotor development was delayed, she achieved autonomous walking after four years of life, and never developed language abilities. On physical examination at two years and three months of age, her length was 85 cm (10–25th p), weight 14 Kg (75–90th centile), CC 46.5 (3rd centile). She was hypotonic. The child presented a behavioral disorder within the autistic spectrum with stereotypies (such as trunk swinging), and sleeping difficulties. EEG registrations and cerebral MRI were normal; severe constipation was reported. She had numerous dysmorphic features including high forehead, strabismus, short nose with markedly anteverted nares, upper lip with marked cupid bow, chubby hands and feet, with bilateral syndactyly of the second and third fingers, chest hypertrichosis (Figure 1B).

At the last examination, at five years of age, she still presented microcephaly and slow rate of growth; absent speech and manual stereotypies were also reported. Gastro-esophageal controls were planned for abdominal pain due to a gall bladder dysmorphism.

Her first diagnostic examinations, which included a complete metabolic workup, conventional karyotyping, array-CGH, Fragile X testing and methylation analysis of the Prader-Willi/Angelman critical region, all showed negative results. Sanger sequencing and real time PCR of the corresponding exonic regions were performed to exclude alterations of the *MECP2* and *GLUT1* genes.

#### 3.1.3. Family 3

Patient 3 is a 17-year-old male born at 39 weeks of gestation from non-consanguineous parents; the mother was 29 and the father was 35 years old. Fetal hydrothorax was observed during pregnancy; the child was born by caesarian section due lack of progression in labor. Neonatal hyperbilirubinemia was corrected with phototherapy for four days. Weight at birth was 3.430 kg (50th centile), length 52 cm (75–90th centile), and OFC 33 cm (<3rd centile).

From the third month of life, the child manifested feeding difficulties and a slow rate of growth, with delayed dentition and closure of fontanelles. Communication problems appeared very early on. Even if social smile was referred at one month of life, the baby did not seem to be interested in people and in the environment; he was frequently agitated, with inconsolable crying and he was intolerant to loud noises. Since infancy, he was hypotonic with deep hyporeflexia; at 13 months of age, he was referred to medical attention for global developmental delay with significant relational difficulties. He sat independently at 36 months; he never crawled and walked independently only at the age of eight years. He started babbling at about six months, but said his first words when he was eight years old.

He always kept a very restricted diet and had difficulties in acquiring masticatory and swallowing functions. Always suffered from meteorism and severe constipation and was toilet trained at 10 years of age. Sleeping difficulties, maintained through childhood, started at about two years of life.

The parents reported occasional and very brief episodes of behavior arrest in early infancy. At 19 months, he presented three focal emotional seizures progressing to bilateral tonic–clonic seizures, in absence of fever. Ictal/inter-ictal EEG recordings resulted normal. At four years of age, he manifested new focal motor seizure during sleep, and his EEG started to show continuous left spikes and waves during sleep. He was diagnosed with ESES at eight years of age and treated accordingly with anti-epileptic drugs. Since infancy, the boy showed behavioral abnormalities, and autistic features with hand stereotypies and repetitive motor patterns (continuously rolling objects and toys), and a reported prevalent happy disposition (Figure 1C).

On physical examination at seven years of age, his height was 109 cm (5–10th p), weight was 16.6 Kg (5–10th p), and OFC was 51 cm (50th p). Some of the dysmorphic features first noticed in infancy became coarser in later years: dolichocephaly, a square-shaped face with a broad forehead, a broad nasal bridge, deep-set eyes with long slightly down slanting palpebral fissures, long eyelashes, bushy eyebrows with mild synophrys, flat philtrum, and thin upper lip (Figure 1C).

The patient, last examined at age 15, presented a better sleeping pattern, improved eating habits and communication skills, although masticatory difficulties, hand stereotypies, and obsessive behavior were still present. He still had hyperacusis, although he better tolerated loud noises. He attended middle school with teaching support; he was quiet and heedful and had a better language comprehension; however, his expressive language was limited to dissyllables. General motility was not fluid and his gait was slow and mildly ataxic. He has been seizure-free since the age of eight, was still receiving antiepileptic therapy and his EEG recordings had improved over the course of the years. The last EEG performed was normal.

Adaptive skills were assessed by the VABS interview, which showed a severe delay in all domains: socialization, communication, daily living, and motor skills (Supplementary Table S1). His first diagnostic examinations, which included a complete metabolic workup, conventional karyotyping and sub-telomeric FISH, array-CGH (100 kb resolution), Fragile X testing, and methylation analysis of the Prader-Willi/Angelman critical region, all showed negative results. Sanger sequencing and real time PCR of the corresponding exonic regions were performed to exclude alterations of the *UBE3A*, *MECP2*, and *CDKL5* genes. Target sequencing of a 31 epilepsy-gene panel was performed to exclude alterations in genes (*GRIN2A, SCN2A, KCNQ2, SLC6A1, SLC9A6, SYNGAP1*, *SHANK3*, and *CNTNAP2*) previously associated with electrical status epilepticus during slow-wave sleep (SES) [16,17].

The father of Patient 3 is a 53-year-old male. His older brother and younger sister were reportedly healthy. No further family history of neuropsychiatric or neurodevelopmental disorders was reported. He had a normal motor development, but experienced learning difficulties since elementary school and was only able to obtain a middle school degree as an adult. Despite no apparent difficulties in writing and reading, a possible dysgraphia, difficulties in mathematics and in abstract reasoning have been reported. He developed practical skills, and at the time of evaluation, he was working as a carpenter.

In childhood, he reportedly manifested hyperactivity, reduced tolerance to frustration, irritability, and reduced sense of danger. He referred good adjustment to the work environment, however, he maintained social relationship only through his wife. At physical examination, mild dysmorphisms were observed such as thin upper lip, elongated palpebral fissures, and synophrys. Spatial orientation seemed to be preserved, but he seemed to have difficulties in correctly indicating the day of the week or the months of the year. A complete neuropsychological examination was performed with the ENB-2 test to evaluate the following cognitive domains: abstract reasoning, language, memory, attention, executive functions, and visual–motor abilities. All of the scores obtained were at the lower normal limit of the scales.

### 3.2. Molecular Data

By targeted gene sequencing, we identified five rare variants of the *WAC* gene, a frameshift deletion, a stop gain, a splice site variant, an in-frame deletion, and a missense variant. The rare missense variant, c.1760G > A(p.Arg587His), and the novel in-frame deletion c.307delAAC (p.Asn103del), were both transmitted by an asymptomatic mother and thus considered of uncertain significance; in particular, the in-frame deletion was found in a male carrying a de novo LoF mutation of *SETBP1*, a pathogenic mutation consistent with his phenotypic manifestations.

**Figure 2:**
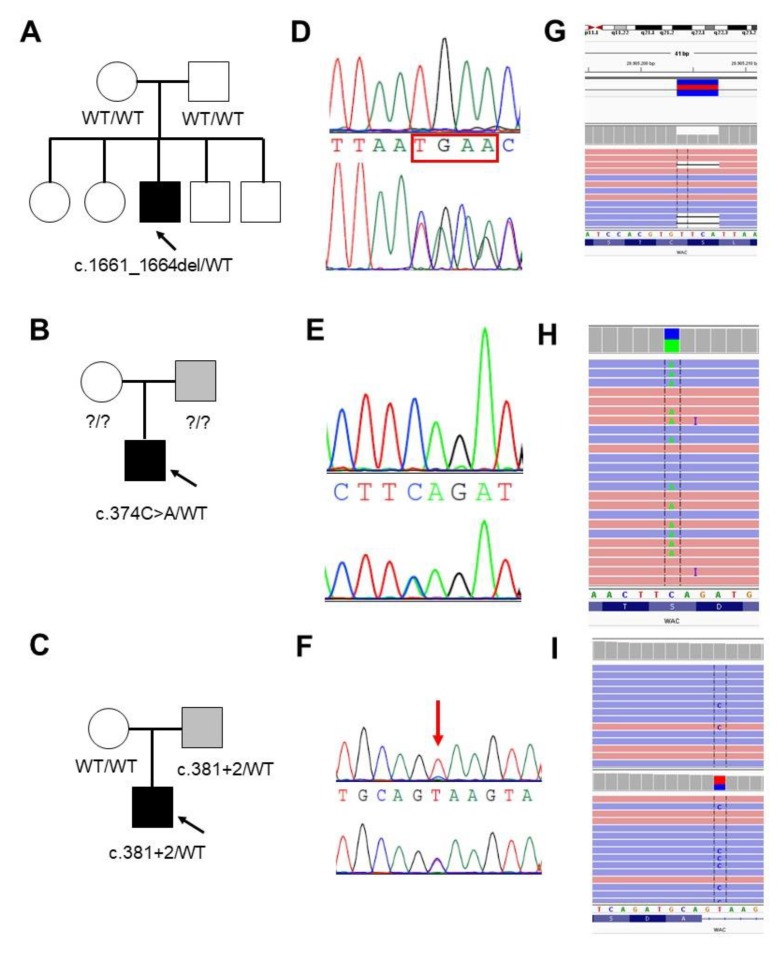
Validation and segregation analysis of the three LoF WAC mutations. (**A**–**C**) Pedigrees of Family 1, 2 and 3, respectively. Probands are indicated with an arrow. (**D**–**F**) Electropherograms of the mutated positions (bottom) compared to the wild-type sequence (upper) (**D**,**E**) or to the mosaic father (upper) (**F**). (**G**–**I**) Screen shot from the Integrative Genomic Viewer (IGV) [18] visualization showing part of the reads at the mutated positions. (**I**) Reads show the presence of mosaicism (43/330; 12%) for the c.381 + 2T > C in the father evaluated in the DNA sample from oral mucosa (upper) compared to the proband (15/21; 42%) (bottom).

The three WAC LoF variants (c.1661_1664del, p.Ser554*; c.374C > A, p.Ser125*; and c.381 + 2T > C), absent from control population (e.g., gnomAD and dbSNP), were validated by Sanger sequencing. The c.1661_1664del (p.Ser554*) and the c.374C > A (p.Ser125*) were previously reported by [8,10]. The frameshift c.1661_1664del resulted to be de novo, while segregation analysis revealed the presence of mosaicism for the c.381 + 2T > C variant in the father of Patient 3 (Figure 2). Targeted next generation sequencing on the father’s blood sample and oral mucosa cells allowed for the estimation of a mosaicism of 16% and 12%, respectively (Figure 2). DNA samples from the parents of Case 2 were not available for segregation analysis.

**Figure 3:**
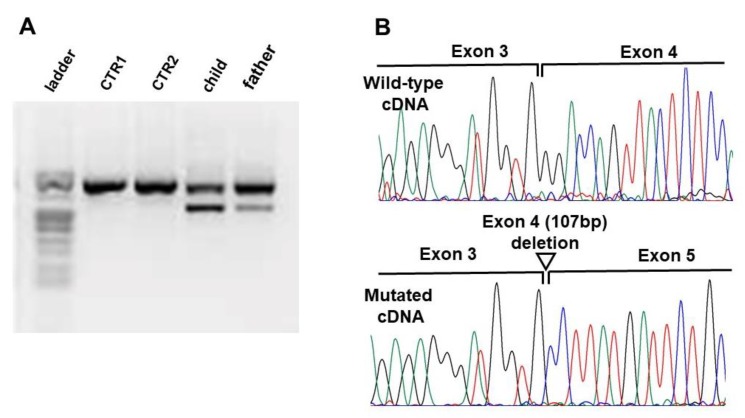
Transcript analysis of the *WAC* splicing mutation identified in Family 3. (**A**) Agarose gel electrophoresis of the Reverse Transcriptase PCR products in the control samples (CTRL1, CTRL2) patient, and his father. (**B**) Electropherograms of the wild-type (upper) and aberrant (bottom) transcript sequence at slicing position showing the skipping of exon 4.

The c.1661_1664del and the c.374C > A variants are both predicted to result in a prematurely truncated protein of residues 554 and 125, respectively, while the c.381 + 2T > C variant in intron four is predicted to have deleterious effects on the donor splice site. To analyze the effect of this latter variant on mRNA splicing, reverse transcriptase-PCR was conducted on RNA extracted from blood leukocytes of the patient and his father. Agarose electrophoresis showed in both samples the presence of a shorter transcript product, with lower intensity in the father sample, compared to the wild type (Figure 3). The Sanger sequencing of this product revealed that the variant causes the skipping of the entire exon 4, resulting in a shorter aberrant transcript predicted to produce a prematurely truncated protein of 93 residues (Figure 3).

### 3.3. WAC Mutation Spectrum in Public Databases

Besides the literature search, we screened public variant databases (LOVD, Clinvar, denovo-DB, and SFARI) to collect all the reported *WAC* pathogenic variants. We counted 58 different pathogenic *WAC* mutations: 29 frameshift, 18 stop-gain, six splice-site mutations, one partial gene deletion, and four missense variants. The majority of them were reported as de novo, while two missense variants identified in subjects with ASD (p.Arg513Gln and p.Thr620Ser) were paternally inherited [19]. One of the two de novo missense variants (p.Ala583Val) was identified in an individual with congenital heart disease (CHD) and no ID; the other (p.Arg613Pro) was reported in a patient presenting clinical features characteristic of *WAC*-related disorder (global developmental delay, constipation, deeply set eye, everted lower lip vermilion, wide mouth [20,21]). The 58 different pathogenic mutations are scattered throughout the gene sequence; no mutation clusters are recognized among exons or domains of the protein. Some of the reported variants have been identified at least in two unrelated individuals, however, a detailed clinical description has been provided only for subjects carrying two of these recurrent mutations: p.Trp574* [8] and p.Arg550* [7] (Supplementary Table S2).

## 4. Discussion

Few individuals with WAC-related ID have been described to date. Due to phenotypic overlap with other neurodevelopmental disorder, it is difficult to establish or even suggest a specific clinical diagnosis of WAC-related ID. Identifying new variants and characterizing individuals with previously described *WAC* mutations will further our understanding of genotype–phenotype correlations and help to highlight the most peculiar features and the amplitude of the WAC phenotypic spectrum.

Gene panel sequencing of 630 individuals with non-specific ID allowed us to identify five rare variants of the *WAC* gene and to establish a certain diagnosis in three novel patients carrying loss of function mutations with WAC-related ID. One of the mutations was inherited from a mosaic father presenting with a milder phenotype. This mutation was identified in an individual with encephalopathy related to electrical status epilepticus during sleep.

### 4.1. WAC Mutations Spectrum

The frequency of pathogenic *WAC* mutations is estimated to be very rare, with a frequency of 3/630 based on our cohort of subjects with developmental delay. All of the reported individuals with the WAC-related disorder carry pathogenic variants predicted to result in a loss of function of the protein and the *WAC* gene is extremely intolerant to LoF variants (pLi = 1) [22]. The de novo frameshift c.1661_1664del, p.Ser554Stop, identified in Patient 1, was recently reported in another individual by [10]. The authors reported a low level of the aberrant transcript in their patient, probably due to nonsense-mediated mRNA decay (NMD). The novel splice site variant c.381 + 2T > C, which was shown to result in a shorter transcript lacking exon 4, is thought to cause loss of normal protein function either through protein truncation or through NMD. Our findings further support the hypothesis that *WAC* haploinsufficiency can be considered the main molecular mechanism implicated in the *WAC*-related disorder. However, the *WAC* gene also seems to be intolerant to variations (Z = 1.51) [3] and few likely pathogenic missense mutations have been identified in patients with ASD, NDDs, and CHD (Supplementary Table S2). The screening we conducted allowed us to also identify a rare missense variant and a novel in-frame deletion of the *WAC* gene (p.Arg587His and p.Asn103del). These variants were considered of uncertain significance, since they were inherited from apparently healthy parents. The in-frame deletion was found in a proband carrying a de novo LoF mutation of *SETBP1,* which was consistent with his phenotypic manifestations. However, we cannot exclude a possible contributing role of this variant in the patient phenotype. Likely pathogenic inherited *WAC* missense mutations have been reported in individuals with ASD, where these can be considered contributing factors [19]. More functional studies and genetic screenings are needed to explore the possible pathogenic role of *WAC* missense variants and their phenotype correlations in order to give the appropriate genetic counselling.

### 4.2. Germinal Mosaicism of WAC Mutations

So far, all the reported individuals with *WAC* LoF mutations have been found to carry de novo variants. DeSanto and colleagues reported two female siblings carrying p.Trp574*, explained only by a germline mosaicism [8]. In this study, Patient 3 inherited the c.381 + 2T > C splice variant from his father, who was found to be mosaic. Paternal mosaicism was estimated in blood and oral mucosa cells to be 16% and 12%, respectively. This can be considered as low-level mosaicism; however, it is difficult to predict the level of mosaicism in brain, since the distribution of abnormal cells can vary widely between different tissues, depending on the timing of the mutational event [23]. To our knowledge, this is the first report of somatic mosaicism for a *WAC* mutation. Mosaicism is increasingly recognized to play a causal role in a variety of human diseases and certainly in neurodevelopmental disorders [24]. Recent studies have shown that de novo mutations in genes important for neurodevelopment, significantly contribute to ASD [25,26,27] and ID [28]. *EHMT1* mosaicism in apparently unaffected parents has been recently found to be associated with ASD and neurocognitive dysfunction [29]. We investigated the cognitive neuropsychological profile of the father of Patient 3 and obtained scores at the lower threshold at all of the tests performed. A non-structured interview allowed us to highlight learning difficulties, especially in childhood, and some weakness in social functioning. Interestingly, he also presented difficulties in the area of memory and temporal orientation, further confirming the possible neuropsychiatric phenotype in apparently asymptomatic carriers of mutations in neurodevelopmental genes, even in the condition of mosaicism.

### 4.3. WAC Phenotype Heterogeneity

The first few cases of *WAC*-related ID were briefly reported from exome/genome sequencing studies of family trios with ID or ASD and, to date, about 60 diverse *WAC* mutations have been reported in public databases (Supplementary Table S2). However, an extensive clinical description has been reported in the literature for only 20 patients [7,8,10,11]. Major features are summarized in Table 1. All described cases presented in early infancy with delayed motor and language development. Intellectual disability, ranging in severity from mild to moderate, were present in almost all reported individuals. Most of the patients presented neonatal hypotonia and behavioral problems with a predominance of ADHD and ASD. Typical facial features are deep set eyes (8/20), long palpebral fessures (9/20), synophrys (7/20), squared shaped face (8/20), bulbose nasal tip (9/20), full lips (8/20), and digital anomalies (12/20). Seizures or epilepsy have been reported in only four patients with WAC point mutations.

Consistent with previously reported cases, our patients showed recognizable craniofacial features (Table 1 and Figure 1). Developmental delay, ID, and behavioral abnormalities also characterized the phenotype of our patients, while hypotonia, feeding difficulties, sleeping disturbances, gastrointestinal and visual problems were not observed in Patient 1. Furthermore, Patient 3 presented epilepsy with a typical EEG pattern of electrical status epilepticus during slow sleep, which has been reported only once [9].

The clinical variability of the *WAC*-related disorder has been observed since the first reported cases and different disease severity has been explained in part by allelic heterogeneity or the mutation position, which can affect different isoforms of the gene or determine whether the transcript is able of bypassing NMD. It has been observed that patients with N-terminal *WAC* mutations are more severely affected when compared with patients carrying C-terminal mutations [8]. This is also the case of Patient 3 carrying the N-terminal mutation, which shows a more severe developmental delay and epilepsy. However, variability of the phenotype has also been observed among individuals carrying the same *WAC* mutation. An extensive clinical description has only been provided for different cases carrying the same p.Trp574* [8] or the p.Arg550* mutation [7]. We observed significant differences by also comparing the phenotype of Patients 1and 2 with those of the individuals reported in the literature, carrying the same *WAC* mutation [8,10]. Patient 1 and the reported patient with p.Ser445* (Patient 1 in [10], both have a global developmental delay, especially a language delay. Their intellectual quotient was estimated in the range of moderate ID and neither were diagnosed as having ASD. However, feeding or gastrointestinal problems were never observed in our patient, while the patient from Uehara and colleagues developed severe constipation from the age of four years [10]. This patient was also diagnosed with growth hormone deficiency at five years of age and developed leukopenia and mild thrombocytopenia at 19 years of age. Our patient, who is currently 16 years old, showed regular physical growth and normal blood count.

Patient 2 and the reported patient with p.Ser125* (Patient 4 in [8]) both have severe developmental delay. Our patient was diagnosed with ASD, but never developed seizures, in contrast to the reported patient.

Variability in disease severity can also be explained by concurrent clinical conditions that can complicate the patient phenotypes such as epilepsy. Seven individuals with *WAC*-related disorder have been reported with seizures: three of them carried a microdeletion of chromosomal region 10p11-p12 [30], and four had a *WAC* LoF mutation [7,8,31]. However, for these individuals, a detailed description of seizures types, and EEG pattern is missing. In this study, we report a LoF *WAC* mutation in a subject with focal epilepsy (patient 3). Similarly, three of the reported individuals with epilepsy carrying a LoF *WAC* mutation had a severe phenotype, which included significant motor and language delay, behavioral problems, sleep disturbances, and gastrointestinal problems.

The role of *WAC* in cognitive processes is supported by functional experiments in *Drosophila*; the *wacky* mutants show a phenotype characteristic of mTOR signaling impairment including slow growth, developmental delay, and increased autophagy [5,7]. Dysregulation of the mTOR pathway has been implicated in several neurological and neuropsychiatric diseases including epilepsy [32,33], Parkinson’s disease, Alzheimer’s disease, cognitive dysfunction, and several syndromes with autistic features [34,35]. The molecular role of mTOR in epileptogenesis has been extensively demonstrated for one of the best-studied mTORpathies, the Tuberos Sclerosis Complex (TSC), in which several mechanisms have been implicated including excitatory/inhibitory synaptic imbalance, leading to hyper-excitability, dysmyelination, impaired autophagy, and induction of immune-proinflammatory pathways [32,36].

Thus, it is not surprising that patients with the *WAC*-related disorder may develop epilepsy. Nonetheless, our patient presented an early onset focal epilepsy (19 months) with status epilepticus during sleep beginning at five years of age and persisting until the age of 14. In ESES, the epileptic activity contributes to worsen cognitive, behavioral, and motor impairment, besides what might be expected from the underlying pathology itself [37]. Interestingly, ESES, has been recently reported in one individual with *WAC*-related ID, however, the details of his phenotype are partially available for comparison [9]. A status epilepticus during sleep has been observed in other syndromes such as Rett syndrome, Christianson syndrome, and other autism spectrum disorders [38,39,40]. Clinical consequences of ESES in neurodevelopmental disorders, especially those characterized by regression, or in neurodegenerative disorders [38], are much more difficult to assess. In the clinical history of our patient, the term encephalopathy was never used although, given the preexisting impairment and our relatively late first assessment, it was not possible to establish the exact timing of his phenotype worsening. Despite a partial rescue of some performances after the resolution of ESES, we believe that encephalopathy, in addition to the WAC-related ID, has contributed to a more severe neurocognitive impairment.

The etiology of electrical status epileptic during sleep is currently under investigation; however, brain malformations, immune disorders, and genetic factors have been implicated. Based on genes found mutated solely in electrical status epileptic during sleep /continuous spikes and waves during sleep/epilepsy-aphasia spectrum, one of the mechanisms involved seems to be an excess of NMDA-transmission [16]. Genetic alterations in genes previously associated to ESES (*GRIN2A, SCN2A, KCNQ2, SLC6A1, SLC9A6, SYNGAP1*, *SHANK3*, and *CNTNAP2*) were excluded in our case [17]. Although other genetic factors could be involved in the electrical status epileptic during sleep pattern in the case we report, the presence of epilepsy and/or electrical status epileptic during sleep might reflect the severe end of the clinical spectrum caused by *WAC* haploinsufficiency. This could be explained by the alteration of mTOR-mediated mechanisms. In brain, in fact, mTOR is involved in neuronal development including neurogenesis and migration, and can influence neuronal excitability by modulating the expression of ion channels and receptors involved in synaptic structure and plasticity [41]. Nonetheless, further investigations are needed to consider electrical status epilepticus during sleep as a constitutive feature of *WAC*-related disorder.

The severity of clinical presentation can only be partially correlated with the molecular causal factors, as it depends on a variety of genetic and environmental factors, and often by their combination. Environmental factors such as maternal lifestyle and premature birth may have variable effects on fetal brain development, resulting in a more severe developmental delay. The sociocultural context in conjunction with health care problems can contribute to the impairment of brain development in an individual with polygenic predisposition [42]. From this perspective, the premature birth and maternal condition during pregnancy in Patient 2 could be contributing factors for the development of a more severe phenotype. Furthermore, the timing in which genetic factors exert their effects depends on the function of the gene involved and on the effects of the mutation (e.g., dosage changes, loss or gain of function) [43]. The impairment of processes involved in brain development such as neuronal proliferation, migration, and maturation may result in different outcomes that can be assessed by neuroimaging techniques and neurophysiological tests [43]. As explained above, the WAC adaptor protein has been implicated in several processes such as transcription elongation, histone H2B ubiquitination regulation, autophagy, and its involvement in the ID phenotype has been demonstrated by studying WAC knockdown in *Drosophila* as a model [2,6,7]. However, further investigations are needed to elucidate the precise neuronal processes impaired in WAC-related ID. This knowledge might help to evaluate the timing of symptoms onset and the possible comorbid conditions that we expect in affected individuals. Most importantly, it would allow for the identification of possible biomarkers pointing toward a specific molecular diagnosis. Collecting the phenotypic details and clinical history of affected individuals, possibly integrated with neuroimaging or neurophysiological tests, is of paramount importance for a better stratification of the clinical presentations in patient cohorts.

In conclusion, here we report three novel individuals with WAC LoF mutations, one of them presenting encephalopathy related to electrical status epilepticus during sleep. These data allowed for expanding the clinical spectrum of WAC-related disorder and suggest that *WAC* should be considered in the genetic screening of individuals with variable ID, behavioral problems, and electrical status epilepticus during sleep or epilepsy. Furthermore, our findings provide further supporting evidence on the possible pathogenic role of mosaic variants on NDDs genes in adult neuropsychiatric features.

## Figures and Tables

**Table 1:** Clinical features of our WAC cases compared to individuals reported in literature.

	Case 1	Case 2	Case 3	De Santo 2015	Lugtenberg 2016	Uehara 2018	Vanegas 2018	Zhang 2019
Sex	M	F	M	5 F, 1 M	6 F, 4 M	3 M	M	F
Age	16 y/o	7 y/o	17 y/o	1.3–11 y/o	1.5–22 y/o	3–22 y/o	4 y/o	5.8 y/o
Normal perinatal period	+	−	+	2/6	6/10	3/3	+	+
Delayed physical Growth	−	+	+	2/6	3/10	2/3	+	−
CC size anomaly	−	+ (<2SD)	+ (<2SD)	N.R.	2/10 (>2SD)	1/3 (>2SD)	+	−
Development								
Intellectual Disability	Moderate	N.E.	Severe	(IQ > 79) 3/6	8/10 (6 Mild, 1 Severe, 1 Moderate)	3/3	+	+
Language delay	+	+	+	6/6	9/10	3/3	+	+
Motor delay	+	+	+	6/6	9/10	3/3	+	+
Behavioral problems								
Autistic features	−	+	+	1/6	4/9	0/3	−	N.R.
Hyperactivity	−	−	+	3/6	4/10	0/3	+	N.R.
Anxiety	−	−	+	3/6	3/10	1/3	+	N.R.
Sleep disturbances	−	+	+	2/6	6/10	0/3	−	N.R.
Stereotypies	−	+	+	1/6	N.R.	0/3	−	N.R.
Neurological								
Hypotonia	−	+	+	6/6	7/9	0/3	+	+
Seizures	−	−	+ (focal)	2/6	1/9	0/3	−	+ (focal)
Epilepsy	−	−	+	1/6	1/9	0/3	−	+
Ocular								
Vision	−	−	+	2/6	5/10	1/3	−	
Strabismus	−	+	−	3/6	3/10	1/3	−	
Dysmorphisms								
Facial shape dysmorphology	−	−	+	N.R.	5/10	2/3	−	+
Prominent forehead	+	−	−	6/6	10/10	1/3	+	N.R.
Bulbous nasal tip	+	−	+	5/6	N.R.	3/3	+	+
Long or downslanting palpebral fissures	−	−	+	1/6	5/10	3/3	−	N.R.
Synophrys	+	−	−	3/6	2/10	1/3	+	N.R.
Deep set eyes	+	−	+	2/6	5/10	0/3	+	N.R.
Full lips - thin upper lip	−	−	−	3/6	N.R.	1/3	−	N.R.
Low-set ears	−	−	−	3/6	N.R.	1/3	+	N.R.
Hirsutism/hypertricosis	−	+	−	2/6	1/10	1/3	+	N.R.
Digital anomalies	−	+	−	1/6	7/7	3/3	+	N.R.
Other								
Feeding difficulties	−	−	+	4/6	4/10	0/3	+	N.R.
Constipation	−	−	+	5/6	N.R.	1/3	−	N.R.
Frequent infections	−	−	−	N.R.	6/8	0/3	+	N.R.
Hearing impairment	−	−	−	2/6	0/2, N.R./8	0/3	+	N.R.
EEG abnormalities	−	−	+	0/2	0/1, N.R./9	N.R.	N.R.	+
MRI abnormalities	−	−	−	1/6	4/9	N.R.	−	−

CC: cranial circumference; IQ: intellectual quotient; NE: not evaluated; NR: not reported; +: present; −: absent.

## Supplementary Materials

The following are available online at https://www.mdpi.com/2073-4425/11/3/344/s1. Table S1: Psychometric test scores for Patient 3, Table S2: WAC mutation spectrum.
